# Control of unsteady laser-produced plasma-flow with a multiple-coil magnetic nozzle

**DOI:** 10.1038/s41598-017-09273-3

**Published:** 2017-08-21

**Authors:** Taichi Morita, Masafumi Edamoto, Satoshi Miura, Atsushi Sunahara, Naoya Saito, Yutaro Itadani, Tomihiko Kojima, Yoshitaka Mori, Tomoyuki Johzaki, Yoshihiro Kajimura, Shinsuke Fujioka, Akifumi Yogo, Hiroaki Nishimura, Hideki Nakashima, Naoji Yamamoto

**Affiliations:** 1 Faculty of Engineering Sciences, Kyushu University, 6-1 Kasuga-Koen, Kasuga, Fukuoka 816-8580 Japan; 2 Interdisciplinary Graduate School of Engineering Sciences, Kyushu University, 6-1, Kasuga-Koen, Kasuga, Fukuoka 816-8580 Japan; 3 Center for Materials Under eXtreme Enviroment (CMUXE), School of Nucelar Engineering, Purdue Uniersity, West Lafayette, Indiana 47907 USA; 4 The Graduate School for the Creation of New Photonics Industries, 1955-1 Kurematsu-cho, Nishi-ku, Hamamatsu, Shizuoka 431-1202 Japan; 5 Faculty of Engineering, Hiroshima University, 1-4-1 Kagamiyama, Higashi-Hiroshima, Hiroshima 739-8527 Japan; 6 Department of Electric and Computer Engineering, Akashi National College of Technology, 679-3 Nishioka, Uozumi-cho, Akashi, Hyogo 674-8501 Japan; 7 Institute of Laser Engineering, Osaka University, 2-6 Yamadaoka, Suita, Osaka 565-0871 Japan

## Abstract

We report an experimental demonstration of controlling plasma flow direction with a magnetic nozzle consisting of multiple coils. Four coils are controlled separately to form an asymmetric magnetic field to change the direction of laser-produced plasma flow. The ablation plasma deforms the topology of the external magnetic field, forming a magnetic cavity inside and compressing the field outside. The compressed magnetic field pushes the plasma via the Lorentz force on a diamagnetic current: **j** × **B** in a certain direction, depending on the magnetic field configuration. Plasma and magnetic field structure formations depending on the initial magnetic field were simultaneously measured with a self-emission gated optical imager and B-dot probe, respectively, and the probe measurement clearly shows the difference of plasma expansion direction between symmetric and asymmetric initial magnetic fields. The combination of two-dimensional radiation hydrodynamic and three-dimensional hybrid simulations shows the control of the deflection angle with different number of coils, forming a plasma structure similar to that observed in the experiment.

## Introduction

Plasma control with an external magnetic field can be practically applied to various areas such as the magnetic control of arc plasma^1^; magnetic confinement of fusion plasma^2^; and the creation of a magnetic nozzle for electric propulsion in a magnetoplasmadynamic (MPD) thruster^3^, helicon plasma thruster^4^, or future propulsion systems such as magnetoplasma sails^5^ and laser fusion propulsion^6, 7^. Thrust vector control (TVC) is a useful technique in thrusters because attitude and/or flight path control are possibly achieved by thrusters themselves. In chemical propulsion, TVC has been successfully achieved by deflecting an exhaust jet by mechanical deflection of a nozzle or thrust chamber, insertion of heat-resistant movable bodies into the exhaust jet, and injection of fuel fluid into the side of the nozzle, or by using separate thrusters^8^. TVC is possibly achieved by deflecting a magnetic nozzle in the case of plasma thrusters. In previous research, especially in the research on laser fusion propulsion, various techniques for thrust deflection have been proposed and studied using numerical simulations: the positioning of the fuel plasma in a magnetic nozzle^9^, the deflection of a coil^9^, magnetic nozzle with multiple coils^10^, and off-axis coils^10^. A system using multiple coils does not need any mechanism of actuating coils, and the magnetic field structure is controlled only by changing the number of coils to drive. Despite this advantage, this technique has never been demonstrated.

In this study, we report the first experimental demonstration of the technique of controlling unsteady plasma flow from laser-ablation with a multiple-coil magnetic-nozzle system. The multiple-coil system forms symmetric or asymmetric magnetic nozzle by driving different numbers of coils. Plasmas produced by laser irradiation expand forming a diamagnetic cavity in the plasma and compressing the field outside, which changes the field topology, and are pushed out via the Lorentz force between the magnetic field and diamagnetic current.

A numerical simulation is performed to analyse the control of plasma flow and the structure formation in this system. The simulation suggests that the structure formed outside the coil is identical to that observed in the experiment, despite the time-evolution being slower than in the experiment. Additionally, the simulation shows the possibility of controlling the plasma flow by changing the number of coils as demonstrated in the experiment.

## Results

**Figure 1:**
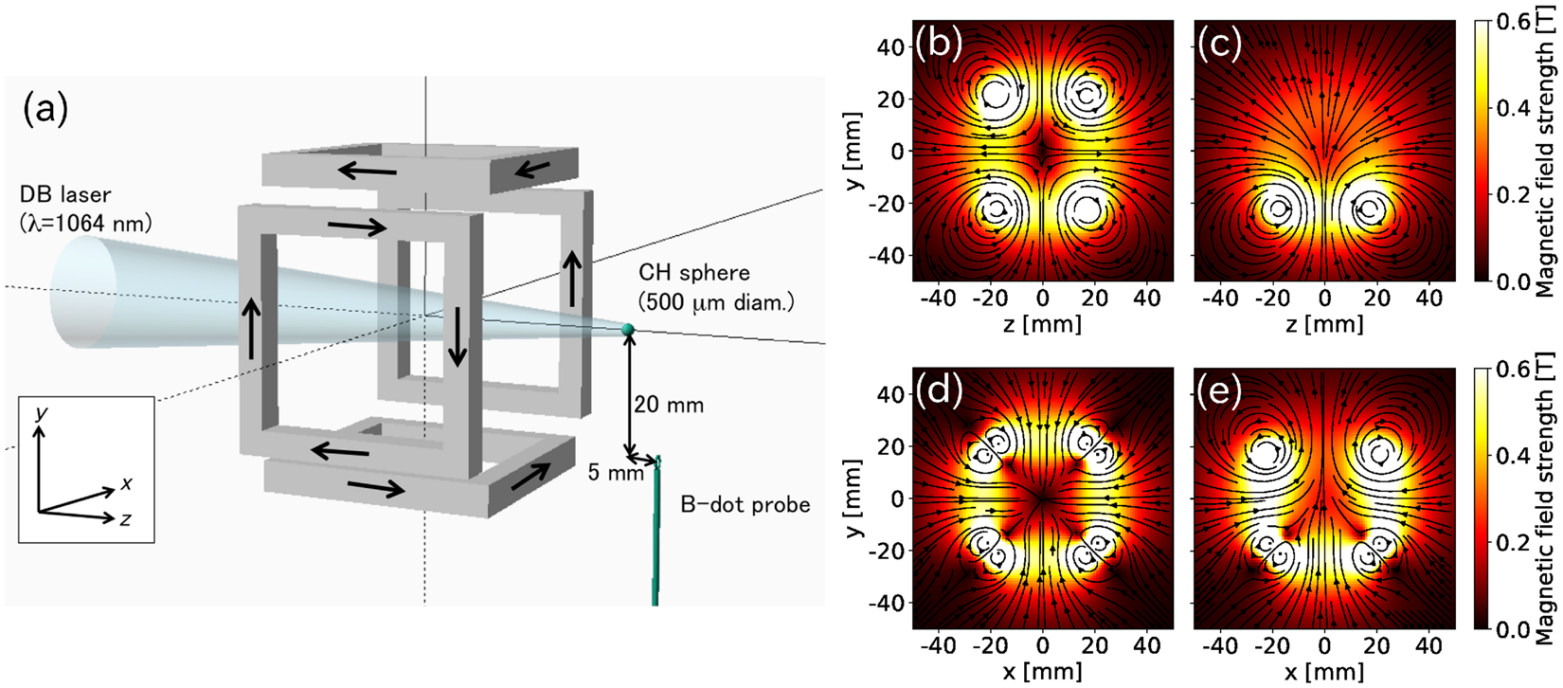
(**a**) A multiple-coil system with four rectangular coils and the experimental setup. A B-dot probe was placed below a spherical target and the target was irradiated with a pulsed laser through the coil. The magnetic field structure used in the experiment on the plane *x* = 0 with (**b**) four and (**c**) three coils, and on the plane *z* = 0 with (**d**) four and (**e**) three coils.

The experiment was performed with an extreme-ultraviolet database laser (DB laser) at Institute of Laser Engineering, Osaka University. A DB laser is an amplified Nd:YAG laser with an energy of 7.5 ± 0.2 J, wavelength of 1064 nm, and pulse duration of 9.4 ± 0.1 ns. A 500 μm diameter spherical CH target was irradiated by the DB laser as shown in Fig. 1(a), through a multiple-coil system. The target was supported by a thin carbon fiber (thickness of approximately 10 μm) attached to a glass stalk with a diameter of 0.9 mm.

This system comprises four eight-turn square coils (30 mm and 38 mm inner and outer side lengths, respectively) located at *x* or *y* = ±21.5 mm. Each coil was individually driven by a pulse-powered circuit consisting of four capacitors (4 × 3 mF), each charged with a voltage of 500 V and driven by laser–triggered gap switches^11^, resulting in a maximum current of approximately 3 kA and a field strength of 0.3 T at the initial target position (32 mm from the center of the coil) by operating all four coils. The duration of the field (approximately 500 μs) is much longer than the plasma expansion time (<10 μs), and the field is quasi-static in the time-scale of plasma expansion. A B-dot probe (single-turn, 2 mm in diameter) was placed 20 mm below the target to measure the time-evolution of the external magnetic field. The inductance of the probe was 10 nH and the impedance of this measurement was 50 Ω, resulting in a time constant of ~0.2 ns, which is much smaller than the time scale of the plasma expansion. The laser-produced plasma initially expands in the −*z*-direction and interacts with the magnetic field to change its direction depending on the field structure. The plasma structure was measured simultaneously by a gated optical imager using an intensified charge-coupled device (ICCD) camera with a minimum exposure time of 5 ns, observing a thermal bremsstrahlung emission at the wavelength of 450 nm (width of 10 nm in FWHM).

The upper and lower panels of Fig. 1 show the magnetic field lines on the planes *x* = 0 and *z* = 0, respectively, with the contours of the field strength, and left and right figures in each panel correspond to the magnetic field structures with all four coils and the lower three coils, respectively. The magnetic field inside the four coil system is cancelled by the anti-direction magnetic field from the counter coils, and it forms a cusp magnetic field at the center of four coils. With the three-coil operation, the field lines diverge at the top while they concentrate at the bottom as shown in Fig. 1(c).

**Figure 2:**
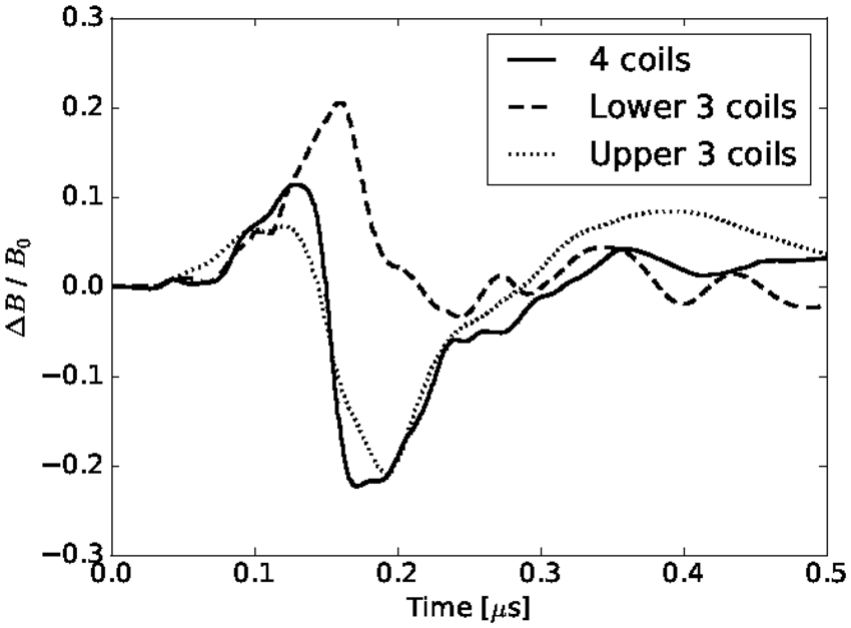
The time evolution of the magnetic field measured with the B-dot probe using all four coils, the lower three coils (top coil is turned off), and the upper three coils (bottom coil is turned off).

Figure 2 shows the time-evolution of the *z*-component of the magnetic field measured with the B-dot probe below the initial target position, as illustrated in Fig. 1(a), in different initial magnetic fields: with the operations of four coils (solid line), the lower three coils (dashed line), and the upper three coils (dotted line). The field increase and decrease in Fig. 2 correspond to the plasma expansion and cavity formation, respectively. As the plasma expands, the field is amplified at *t* ~ 0.1–0.15 μs under all three operating conditions. The field strength suddenly decreases after its peak and becomes even smaller than the initial values with the operation of all four coils and the upper three coils. The field, however, does not decrease in the case of the lower three coils, suggesting that the plasma expands upwards and does not reach the probe.

**Figure 3:**
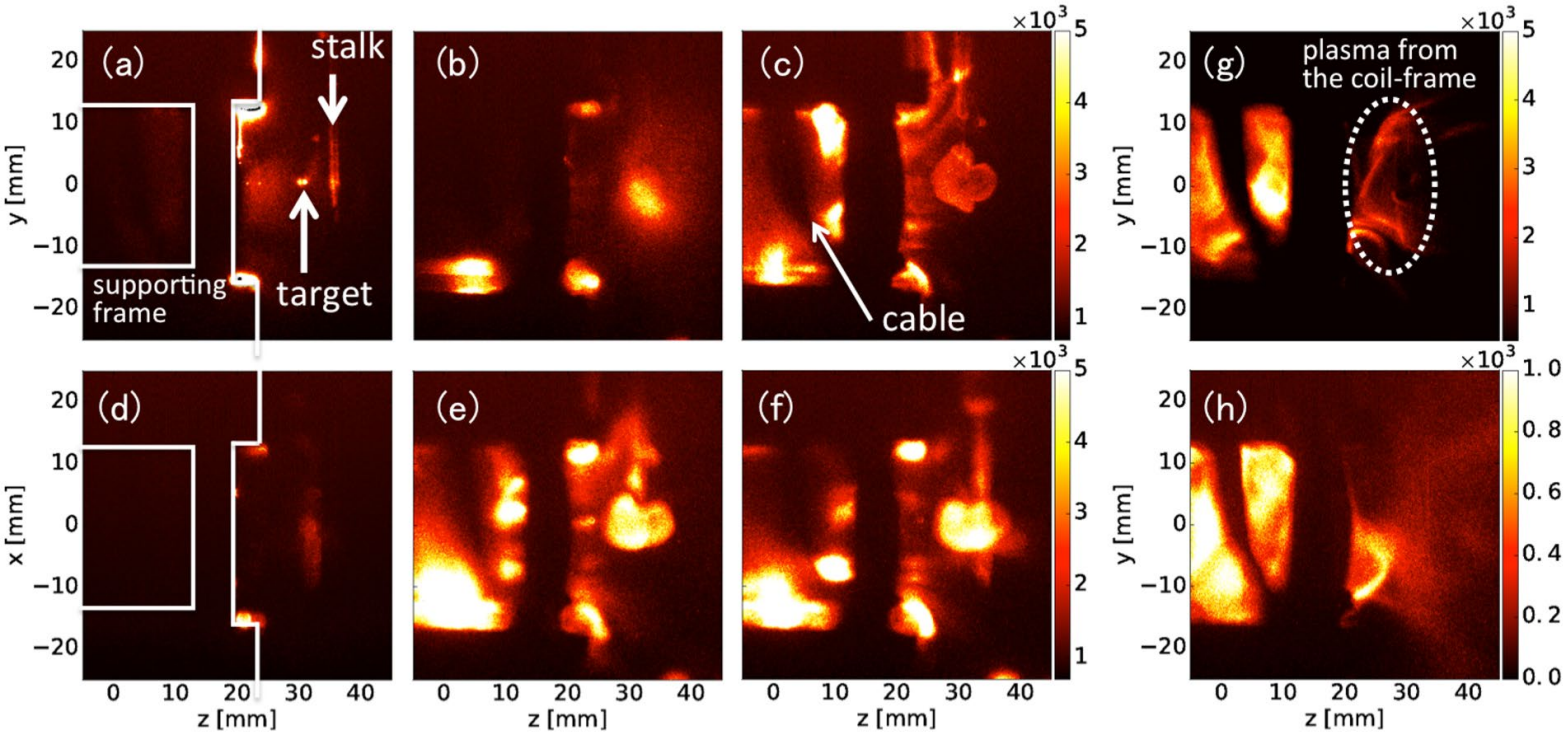
Self-emission at *t* = 0.1 μs [(**a**) and (**d**)], 0.2 μs [(**b**) and (**e**)], and 0.5 μs [(**c**) and (**f**)]. Upper and lower panels show, respectively, the data obtained from four-coil and three-coil operations. White lines show the surface of a supporting frame of the coil. The data (**g**) and (**h**) are the emissions at 2 μs and 5 μs, respectively, with four coils.

Figure 3 shows the plasma emission at different times. Panels (a)–(c) and (d)–(f) show, respectively, the emission with four-coil and lower-three-coil operations, at *t* = 0.1 μs [panels (a) and (d)], 0.2 μs [(b) and (e)], and 0.5 μs [(c) and (f)]. The surface of the supporting frame of the coil is depicted with white lines. The DB laser irradiates the CH sphere at *t* = 0, producing a plasma that is expanding leftwards, as shown in Fig. 3(a) and (d). A glass stalk and the surface of the frame are ablated as well by radiation from the plasma and bright emissions are observed at *z* ~ 40 mm and 20 mm, respectively. The plasma is decelerated by the magnetic field, and high-density plasma exists at *z* = 30–40 mm, as shown in Fig. 3(b) and (e) at *t* = 0.2 μs and in Fig. 3(c) and (f) at *t* = 0.5 μs, forming a cone-like structure. Additionally, a part of the expanding plasma enters the coil system as shown at *t* > 0.2 μs. In addition, later in time as shown in Fig. 3(g) (*t* = 2 μs) and 1(h) (*t* = 5 μs), plasmas inside the coil system continue emitting longer than 5 μs.

The plasma expansion with the multiple-coil system was also analysed with numerical simulations. The laser absorption and plasma generation were simulated with a two-dimensional radiation hydrodynamic code Star2D^12^ for 33 ns (until 8 ns after the laser peak) without considering an external magnetic field. This code uses a one-fluid and two-temperature model, and ions are treated as the average of carbon and hydrogen. The plasma expansion in the external magnetic field was simulated with a three-dimensional hybrid code^7^, in which ions and electrons are treated as individual super-particles and electromagnetic fluid, respectively, in the magnetic field with four [Fig. 1(b) and (d)], three [Fig. 1(c) and (e)], and two (not shown) adjacent coils. The total number of super-particles was 10^6^ and they were distributed according to the ion density calculated by the radiation hydrodynamic simulation.

**Figure 4:**
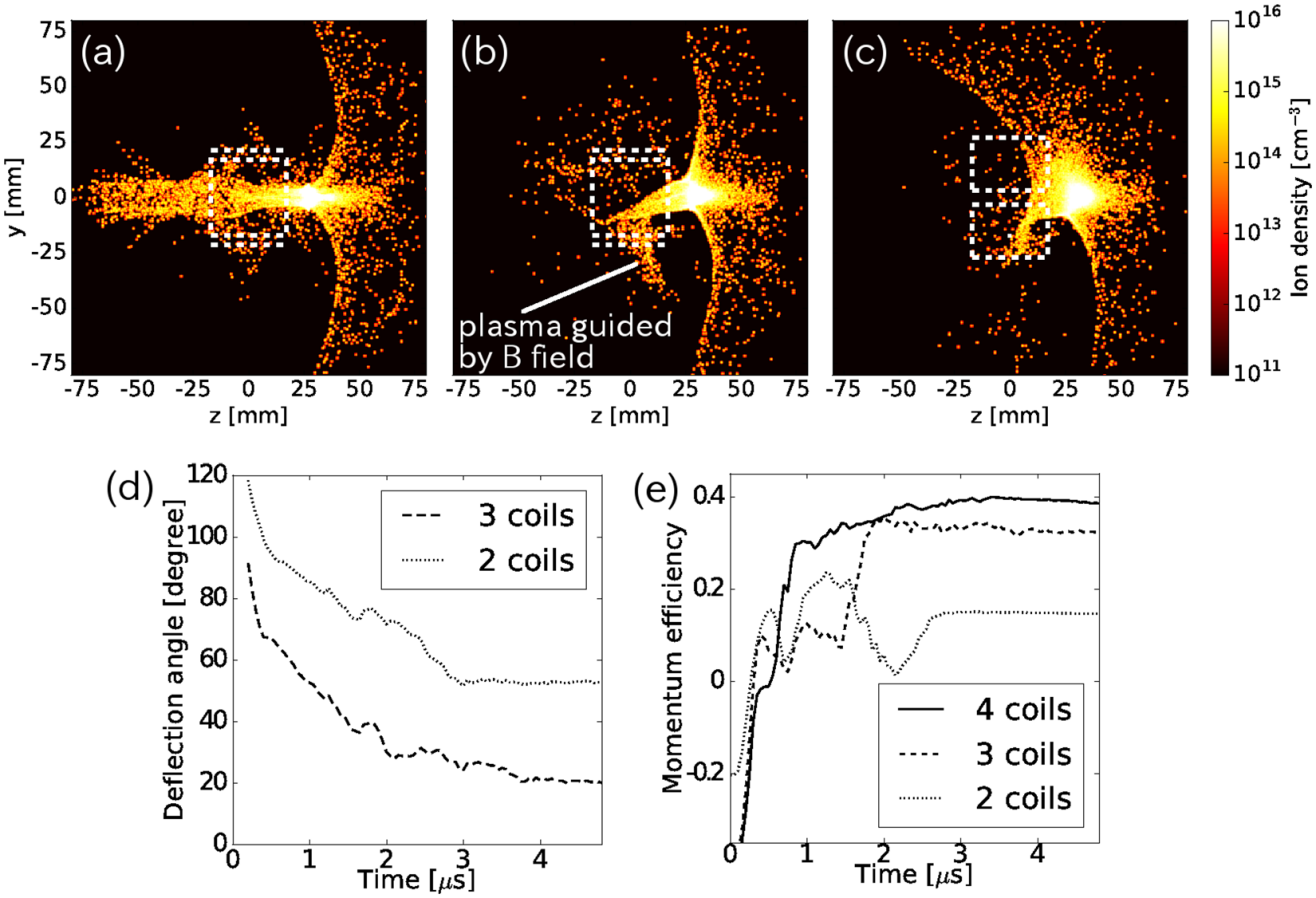
Simulation results from a combined calculation of two-dimensional radiation hydrodynamic and three-dimensional hybrid simulations. Ion density distribution in the unit of cm^−3^ on the plane *x* = 0 with (**a**) all four coils, (**b**) the three lower coils, and (**c**) two coils at *t* = 0.5 μs. Time-evolutions of (**d**) the deflection angle: $\theta ={\tan }^{-1}({\rm{\Sigma }}{v}_{y}/{\rm{\Sigma }}{v}_{z})$ and (**e**) momentum efficiency: ${\rm{\Sigma }}{m}_{i}{v}_{\theta }/{\rm{\Sigma }}{m}_{i}|v|$.

Figure 4(a–c) show the ion density distributions estimated from the super-particle distributions with the four, lower three, and lower two coils, respectively, at *t* = 0.5 μs. The coil positions are illustrated as dashed lines in the figures, and in the case of two coils, the coils are positioned as 45 degrees rotated about the *z*-axis, to see the maximum deflection, as depicted in Fig. 4(c). The laser irradiates the target from the left side and the laser-produced plasma initially expands leftwards. The plasma interacts with the magnetic field being directed to rightwards, as shown for *z* > 20 mm in the three figures, while a part of the plasma enters the coil system and flows out through the coils, as shown in top and/or bottom regions in Fig. 4(a–c). In the cases of two and three coils, high-density plasmas are observed near the cusp region at the bottom coil where the magnetic field becomes stronger [Fig. 4(b) and (c)] as observed in the experiment [Fig. 3(c) and (f)], and low-density plasma expands upwards.

**Figure 5:**
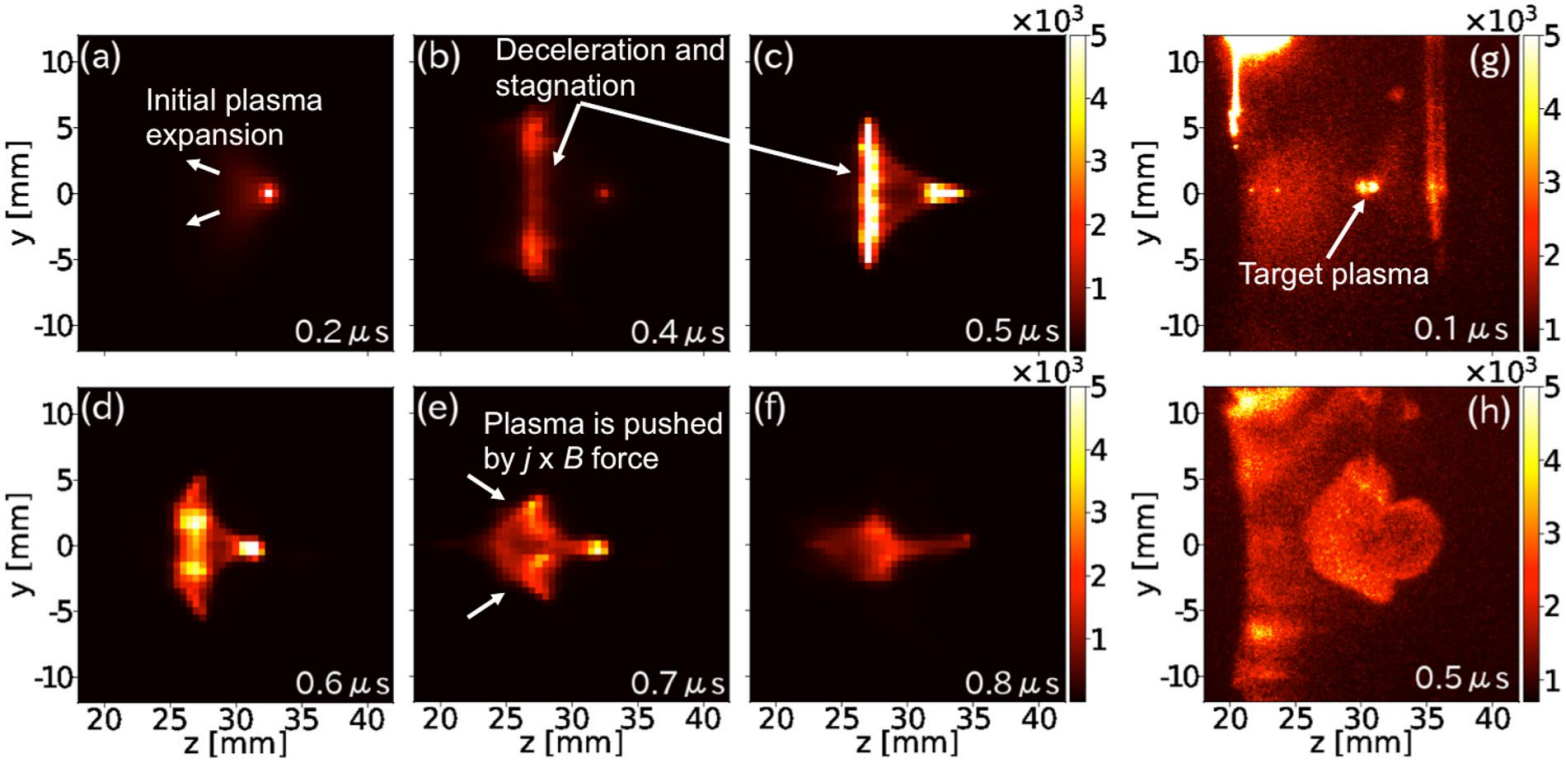
Artificial camera images generated from numerical simulation with four-coil operation at (**a**) *t* = 0.2 μs, (**b**) 0.4 μs, (**c**) 0.5 μs, (**d**) 0.6 μs, (**e**) 0.7 μs, and (**f**) 0.8 μs outside of the coil. The camera images at (**g**) *t* = 0.1 μs and (**g**) 0.5 μs obtained from the experiment are shown as well.

The emission structure observed in the experiment is estimated from the simulation to understand the time-evolution and structure formation of the expanding plasma. In general, the thermal bremsstrahlung emission strongly depends on the ion number density and charge states, and weakly on the temperature^13, 14^, as shown in equation (1). Here, artificial camera images are generated in terms of the line-of-sight integration of the emission energy in arbitrary units as $I\propto \int {n}_{i}^{2}{Z}^{3}dl$, where *I*, *n*_*i*_, and *Z* are the brightness, ion number density and charge state, respectively, and shown in Fig. 5(a–f) as the time-evolution from *t* = 0.2 μs to 0.7 μs. Figure 5(g) and (h) show the self-emission data near the target position [see Fig. 3(a) and (c)]. The laser-produced plasma, which is expanding leftwards, stagnates at *z* ~ 25 mm, as shown in Fig. 5(b) (*t* = 0.4 μs) and (c) (*t* = 0.5 μs) resulting from interaction with the magnetic field. Later in time, the plasma is pushed via the **j** × **B** force and forms a cone-like structure, as shown in Fig. 5(d) (*t* = 0.6 μs), (e) (*t* = 0.7 μs), and (f) (*t* = 0.8 μs).

## Discussion

The magnetic skin depth of the laser-produced plasma is estimated from a similar experiment with the same laser parameters^15^ as $c/{\omega }_{pe} \sim 5.3$ μm and is much smaller than the typical plasma size of a few tens of millimetres. Moreover, the diffusion time of the magnetic field^15^ is *τ* ~ *l* ^2^/*η* ~ 50 μs with the plasma size of 10 mm, meaning that the plasma in this experiment is highly conductive, and therefore, the plasma expands producing a magnetic cavity inside itself and amplifying the field outside.

As the magnetic field measurement shows, under all conditions, the cavity depth is small: ${\rm{\Delta }}B/{B}_{0} \sim 0.2$ at *t* ~ 0.2 μs, as shown in Fig. 2, that is, the magnetic field is not fully expelled. The magnetic cavity size can be estimated in an expanding plasma as the total excluded magnetic energy, which is equivalent to the plasma kinetic energy inside^16–18^: ${R}_{b}={(3{\mu }_{0}{E}_{{\rm{lpp}}}/2\pi {B}_{0}^{2})}^{1/3}$, where *μ*_0_ is a magnetic permeability in vacuum, ${E}_{{\rm{lpp}}}$ is the kinetic energy of the laser-produced plasma, and *B*_0_ is an external magnetic field. In the present experiment, the maximum *R*_*b*_ is approximated, assuming 100% of the laser energy is converted to plasma kinetic energy^19^, as *R*_*b*_ ~ 48 mm, which is twice as large as the distance between the initial target position and the probe. In previous research^18, 20, 21^, the plasma expansion radius *R*_*p*_ was found to be ${R}_{p} \sim 0.5{R}_{b}$, and it is comparable to the distance of the probe. Moreover, as discussed in ref. 21, the cavity depth increases with decreasing external magnetic field. This finding suggests that the plasma kinetic energy is small compared with the magnetic field energy.

The emission intensity at the cusp region in the bottom coil (*z* ~ 0 and *y* ~ −15 mm) is stronger than that at the center in both cases as indicated at *t* = 0.2 μs [Fig. 3(b) and (e)] and *t* = 0.5 μs [Fig. 3(c) and (f)]. Additionally, the cusp region with three coils shows stronger emission than that with four coils. In the case of three coils, the plasma inside is guided downwards along the magnetic field lines [see Fig. 1(c)] and the density at the bottom is higher than that with the case of four coils. This deviation on the plasma density is also observed in the numerical simulations as shown in Fig. 4(a–c). As time passes, although the density decreases, the plasma propagates rightwards more than 5 μs for *z* > 25 mm as a result of the Lorentz force. Comparing the plasma structures in the simulation and experiment early in time [Fig. 5(a) and (g)], and later in time [Fig. 5(e) and (h)], the simulation well replicates the plasma structure, such as the stagnation position of *z* ~ 25 mm, plasma expansion scale, and cone-like shape later in time, even though the time-evolution in the simulation is approximately 0.1–0.2 μs slower than in the experiment. This difference in time can be caused by some assumptions in our simulation: the assumption of average ion mass (6.5 *m*_*p*_ from carbon and hydrogen, where *m*_*p*_ is the proton mass), without ionization and recombination, and the cold plasma assumption (*T*_*e*_ = *T*_*i*_ = 0) in the hybrid simulation; and/or plasma physics conditions that are not included in our simulation codes: the external magnetic field in the radiation hydrodynamic simulation, ablation of the supporting frame, and interaction between plasma flows.

Deflection angles are estimated from the simulations as $\theta ={\tan }^{-1}({\rm{\Sigma }}{v}_{y}/{\rm{\Sigma }}{v}_{z})$, where the summation is taken over super-particles in the flow outside of the coil system, and the deflection relative to that in four–coil operation is shown in Fig. 4(d) in two cases: with the operations of three and two coils. Here, the maximum angles of 56.7 degrees and 20 degrees are obtained from the operations of two adjacent coils and three coils, respectively. However, as discussed in previous numerical simulations^9, 10^, large deflection may result in a low momentum efficiency because the plasma diverges in weaker magnetic fields and the velocity distribution in a perpendicular component ${v}_{\theta \perp }$ broadens, colliding with the coils, as Fig. 4(b) and (c) suggest. Here, the momentum efficiency is expressed as $\eta ={\rm{\Sigma }}m{v}_{\theta \parallel }/\Sigma m|v|$, where ${v}_{\theta \parallel }$ is the velocity along the deflection angle *θ*, and *θ* = 20° and 56.7° with three and two coils, respectively. Figure 4(e) shows the time–evolution of the momentum efficiency in three cases: four–, three–, and two–coil operations. Initially, the plasma expands in the -*z*–direction, meaning the efficiency *η* is negative. As the plasma is pushed by the magnetic field, *η* increases and *η* > 0, as shown after *t* > 0.3 μs. With a large deflection of 56.7 degrees (two coils), the efficiency (~0.14) is much smaller than in the other two cases, and the configuration of multiple coils should be optimized to increase the efficiency and avoid damage to the coils from the collision. As the present simulation suggests, the deflection is difficult measure with imaging diagnostics, as shown in Fig. 3. Further diagnostics such as measurements of the ion density distribution and plasma flow velocity and the direct measurement of a repulsive force on a coil along two axes will be required.

In summary, we demonstrated a multiple-coil magnetic nozzle system to control unsteady laser-produced plasma flow. The plasma flow was deflected in a certain direction depending on the initial magnetic field structure, which could be altered by changing the number of coils being operated. The magnetic cavity and field compression were observed with a B-dot probe, and the field structure changed depending on the flow direction. A combined numerical simulation of two-dimensional radiation hydrodynamic and three-dimensional hybrid codes shows consistency with the experiment in terms of the density structure inside and outside the coil system. The structure outside this system is evaluated using artificial camera images from the simulation and compared with that observed in the experiment. Though the time-evolution in the simulation is ~0.1–0.2 μs slower than the experiment, the plasma structures both from the experiment and simulation are identical. The simulation suggests that the maximum deflection angles of 56.7 degrees and 20 degrees are obtained with the operations of two coils and three coils, respectively, in the same setup as in the experiment.

## Methods

### Experimental Details

The DB laser is an amplified single–pulse Nd:YAG laser, and the experimental data with different magnetic configurations were obtained in different laser shots. The reproducibilities of energy and pulse width were well within approximately 3% and ~1%, respectively, and the plasma expansion and structure formation were obtained as shown in Fig. 3.

The plasma structure was measured with a gated optical imager at a wavelength of 450 nm with a width of 10 nm in FWHM, in which no strong emission lines exist for carbon and hydrogen atoms. The plasma density in the present experiment is normally optically thin, and the emission is evaluated as thermal bremsstrahlung emission.

1 $$\begin{array}{rcl}{\varepsilon }_{\lambda }({T}_{e}) & = & \frac{{2}^{5}\pi {e}^{6}}{{(4\pi {\varepsilon }_{0})}^{3}3m{c}^{2}{\lambda }^{2}}{(\frac{2\pi }{3m})}^{1/2}{g}^{ff}\frac{{Z}^{2}{n}_{e}{n}_{i}}{\sqrt{{T}_{e}}}\exp (-hc/\lambda {T}_{e})\\  & \propto  & {Z}^{3}{n}_{i}^{2}\exp (-hc/\lambda {T}_{e}){g}^{ff}/\sqrt{{T}_{e}},\end{array}$$

where *e* is the elementary charge, *ε*_0_ is the permittivity of free space, *c* is the speed of light, *m* is the electron mass, $\lambda \simeq 450$ nm is the wavelength, *h* is the Planck constant, and ${g}^{ff} \sim 1$ is a velocity averaged Gaunt factor^22^. The emission intensity, therefore, strongly depends on the electron density and charge state, and the experimental and simulation results shown in Fig. 5 can be directly compared.

### Simulation Methods

To simulate the laser-produced plasma, we conducted the two-dimensional radiation hydrodynamic simulation^12^ without an external magnetic field in an early stage of the plasma expansion. The laser pulse is in Gaussian shape in both time and space, and is propagated with the ray-tracing technique. The laser peak is set at *t* = 25 ns and the simulation ends at *t* = 33 ns.

The density distribution was converted to ion particle and electron fluid density and given as input to the three-dimensional hybrid simulation^7^, in which the equations of motion for electrons and ions, the equation of internal energy of electron fluids, and Maxwell equations are self-consistently calculated in the magnetic field as shown in Fig. 1(b–e) with four–, three–, and two–coil operations. In this calculation, the cold plasma condition (*T*_*e*_ = *T*_*i*_ = 0) is assumed for simplicity.

### Data Availability

The datasets generated and/or analysed during the current study are available from the corresponding author on reasonable request.
